# Goal Fluency, Pessimism and Disengagement in Depression

**DOI:** 10.1371/journal.pone.0166259

**Published:** 2016-11-30

**Authors:** Joanne M. Dickson, Nicholas J. Moberly, Christian O’Dea, Matt Field

**Affiliations:** 1 Department of Psychology, Edith Cowan University, Joondalup, Australia; 2 Department of Psychological Sciences, University of Liverpool, Liverpool, United Kingdom; 3 Mood Disorders Centre, University of Exeter, Exeter, United Kingdom; 4 Newhall Independent Hospital, Mental Health Care, Wrexham, United Kingdom; Rijksuniversiteit Groningen, NETHERLANDS

## Abstract

Despite the development of prominent theoretical models of goal motivation and its importance in daily life, research has rarely examined goal dysregulation processes in clinical depression. Here we aimed to investigate problematic aspects of goal regulation in clinically depressed adults, relative to controls. Depressed participants (*n* = 42) were recruited from two Improving Access to Psychological Therapy clinics in north-west England. Control participants (*n* = 51) were recruited from the same region. Participants generated personal approach goals (e.g., *improve my marathon time*) and avoidance goals (e.g., *avoid getting upset over little things*) and completed self-report measures of goal attainment likelihood and depressive symptoms. Participants also completed a measure of ease of disengagement from unattainable goals and re-engagement with new goals. Compared to controls, depressed participants reported fewer approach goals (but not more avoidance goals), rated their approach goal (rewarding) outcomes as less likely to happen and avoidance goal (threatening) outcomes as more likely to happen. Depressed participants also reported greater ease of disengagement from unattainable goals and more difficulty re-engaging with new goals than controls. Our findings extend current knowledge of the psychopathology of depression from a goal regulation perspective, suggesting that pessimism around goal pursuit accompanies fewer approach goal pursuits and a general tendency to disengage when difficulties are encountered.

## Introduction

Prominent theories of motivation posit that dysfunctions in approach and avoidance systems underpin emotional susceptibility and affective disorders [1– 2]. Gray [3] proposed a behavioural activation system (BAS) that is sensitive to reward stimuli and a behavioural inhibition system (BIS) that is sensitive to threat stimuli and triggered by approach-avoidance conflict. Fowles [4] characterized depression in terms of low BAS and high BIS sensitivities. Consistent with this, depressed individuals are less responsive to reward stimuli than controls [5–6] and EEG studies have characterized depression in terms of decreased activity in left prefrontal regions that regulate approach motivation [1]. Self-report measures also suggest that depression is characterized by reduced BAS activity and increased BIS activity [7].

Research has begun to study depression in terms of personal goals: idiographic motivational representations that guide sustained activity and are fundamental to human experience [8–9]. Consistent with a two-system model of motivation, approach goals refer to representations of desirable future goal outcomes (e.g., *‘to run a marathon’*) whereas avoidance goals refer to representations of undesirable future outcomes (e.g., *‘to avoid letting little things upset me’*) [10–11]. Emerging empirical evidence implicates goal dysfunction in depression [9], [12–13].

Past research using adolescent non-clinical samples has supported Fowles’ theoretical assumptions [14–15]; however, studies using adult clinical samples reveal mixed results. Some studies show a deficit in approach goal motivation in depression [16] while others do not [17–18]. Findings supporting increased avoidance goal motivation in clinical depression are also mixed [19–20]. Many preliminary clinical studies have relied on small sample sizes with adequate power to detect large effect sizes only, thus limiting conclusions from null findings [17–18]. In this study, we recruited a larger sample to more adequately address Fowles’ hypothesis in the context of personal goals.

This study also examined the ability to flexibly adjust goals in depression, which is considered important to motivation and mental health [21]. Although a couple of studies have investigated self-regulation of unattainable goals in suicide attempters [22–23], to our knowledge no research has yet investigated self-regulation of unattainable goals in clinical depression. Pyszczynski and Greenberg’s [24] self-regulatory perseveration theory posits that the continued pursuit of an unattainable goal may create a ‘spiral’ into depression involving excessive self-focus and negative affect. Many theorists have also suggested that psychological difficulties are maintained by inflexible goal processes [25–27]. Wrosch and colleagues [28–29] proposed that goal adjustment abilities are underpinned by two distinct abilities: goal disengagement and goal re-engagement. Goal disengagement is thought to relieve psychological distress by reducing commitment and withdrawal of effort towards an unattainable goal, preventing prolonged distress. When an important goal has been abandoned, goal re-engagement can orient the individual to alternative goal(s) and increase positive aspects of subjective wellbeing [30].

Individual differences in adjusting to unattainable goals are thought to represent a vulnerability marker for depression [10]. Faced with an unattainable goal, arguably the most adaptive response is to withdraw effort and commitment from it [10, 31], whereas continued pursuit of unattainable goals may reduce self-regulatory efficiency and increase risk of depression [17]. Researchers have hypothesized that both impaired goal disengagement and impaired ability to re-engage with new goals characterize depression [17, 32]. Furthermore, Wrosch, Amir and Miller [30] suggest that engagement with new goals may be compromised if an individual withdraws effort but remains committed to an unattainable goal.

The ability to flexibly disengage from goals and engage with new goals is likely to be related to expectancies of goal attainment. Goal expectancy is central to motivation [33] and is proportional to the effort and commitment that an individual exerts towards achieving goals in the face of obstacles [10, 34]. Previous research has found that pessimism in depression extends to personal approach and avoidance goals [17]. Low expectancies generally accompany a disengagement tendency [10]. However, in depression, reduced expectancies for goals may be accompanied by reduced flexibility that engenders a state of ‘painful engagement’ [32]. At the same time, inflexible goal adjustment and reduced motivation to engage in rewarding activities in depressed individuals may compromise engagement in new goals [35].

In this study, we therefore hypothesized that depressed individuals, relative to never-depressed controls, would report (i) fewer approach goals and (ii) more avoidance goals, (iii) lower expectancies for desirable future approach goal outcomes, (iv) higher expectancies for undesirable avoidance goal outcomes, (v) lower goal disengagement in response to an unattainable goal, and (vi) lower re-engagement in new goals in response to an unattainable goal.

## Method

### Participants

Clinical participants were recruited from two National Health Service (NHS), Improving Access to Psychological Service (IAPT) sites in North West England. Participants were accessing a low intensity cognitive behavioural therapy treatment, delivering goal-based approaches to depression (e.g., behavioural activation). Control participants were recruited from the community within the same region. Advertisements and leaflets regarding the study were distributed in local gyms, churches, gyms and social groups.

#### Depressed group

Forty-two participants (25 women, 17 men; age 16–67 years, *M* = 38.5, *SD* = 13.7) met the American Psychiatric Association study criteria for depression [36]. Depressed participants were recruited directly from two IAPT clinics. Service users meeting criteria were invited to participate by NHS staff undertaking therapy sessions at the assessment stage. Pre-test screening for depression was conducted at the respective IAPT services. For the purposes of this study, a brief structured interview was designed and the Personal Health Questionnaire (PHQ-9) [37] was used to assess depressive symptomatology. The brief interview schedule assessed items such as number of previous depressive episodes, prescribed medication for depression (see Table 1 for details). The PHQ-9 incorporates DSM-IV diagnostic criteria for depression and is used for screening, diagnosing, monitoring and measuring the severity of depression [37**]**. The brief interview and PHQ-9 were administered to all participants. Depressed participants met DSM-IV criteria for depression as the primary interfering condition based on (i) assessment by a clinical psychologist, (ii) and PHQ-9 scores in the symptomatic range on depression (scores ≥ 9). All participants were aged 16 years or older and at the assessment stage of their therapy. Consistent with DSM-IV, exclusion criteria included substance abuse, psychotic symptoms, bipolar disorder, head injury, and mood disorder due to a general medical condition. Participants were also excluded if they reported current suicidal ideation and/or were not fluent in English.

**Table 1:** Clinical characteristics of the depressed group (*n* = 42).

	Number	%
**PHQ-9 classification**		
Mild	7	17%
Moderate	17	40%
Moderate to severe	15	36%
Severe	3	7%
**Previous therapy**		
None	25	60%
One	14	33%
Two	3	7%
**Type of therapy**		
None	25	60%
Counselling	12	29%
Cognitive Behavioural Therapy (CBT)	2	5%
Counselling and CBT separately	1	2%
Counselling on two occasions	2	5%
**Previous depressive episodes**		
None	20	48%
One	11	26%
Two	9	21%
Three	1	2%
Four	1	2%
**Medication**		
Yes	7	17%
No	35	83%

#### Control group

The control group comprised 51 participants (32 female, 19 male; age 21–61, *M* = 36.3, *SD* = 13.4) recruited in the same geographical region and matched as closely as possible to depressed participants on demographic variables (e.g., age, gender, socio-economic status). Inclusion in the control group required that participants (i) reported never having a depressive disorder or other psychological disorder, (ii) scored within the asymptomatic range on the PHQ-9 (scores < 9; *M* = 1.83, *SD* = 2.17, range = 0–8), (iii) had never accessed services, (iv) were not currently receiving support for mental health difficulties, including medication or psychotherapy, and (v) were aged 16 years and over.

### Materials

**Goals task** [14]. In separate periods, participants are instructed to list approach and avoidance goals that they think will characterize them in the future (e.g., next week, next month, in a few years) using short single statements. Goals are described as future experiences that they will be trying to accomplish (e.g., ‘to take a summer holiday with friends’) or to avoid (e.g., ‘to not upset my family’). Prompts are provided to elicit approach goals (‘*It will be important for me to…*’) and avoidance goals (‘*It will be important for me to avoid…*’). To reduce variation associated with effort, participants are given 90 seconds to write down as many personal goals that come to mind in each goal condition (approach and avoidance). The order of the approach and avoidance goal measures was counterbalanced.

Following data collection, each goal was coded as approach or avoidance by an author (CO) and an independent rater, yielding complete agreement (*k* = 1). Across participants, sixteen avoidance goals were listed in the approach goal condition and three approach goals were listed in the avoidance condition. These goals (<1%) were excluded from the count, although their omission did not affect the results.

#### Goal importance [14]

Participants rated the subjective importance of each goal on a one-item Likert-type scale from 1 (*not very important*) to 9 (*extremely*).

#### Goal expectancy [38]

Expectancy judgements for each approach and avoidance goal outcome were rated on a Likert-type scale from 1 (*not at all likely to happen*) to 9 (*extremely likely to happen*). In the avoidance condition, a higher score corresponded to greater likelihood of *failing* to avoid the unwanted outcome.

#### Goal Adjustment Scale (GAS) [29]

The GAS measures individual differences in goal disengagement (four items) and goal re-engagement (six items) tendencies in response to unattainable goals. Participants rate the extent to which they engage in various behaviours when they are unable to attain important goals, using a five-point Likert-type scale from 1 (*strongly disagree*) to 5 (*strongly agree*). Higher scores indicate greater ability to disengage (GAS-D) from unattainable goals and to re-engage in alternative new goals (GAS-R). The measure has good reliability and validity [29]. In the present study, Cronbach’s alpha was .66 for goal disengagement and .89 for goal re-engagement.

#### Written fluency task [39]

Participants completed an abbreviated form of the FAS task to familiarise participants with writing under time pressure and to assess written fluency. Participants were instructed to write down as many words as possible beginning with the letter ‘F’ within 90 seconds [17]. Groups did not differ significantly on written fluency (*p* > .05).

#### Personal Health Questionnaire, PHQ-9; [37]

The PHQ-9 is a nine item self-report measure used to assess the presence and severity of depressive symptoms. Participants rate how often each of the depressive symptoms has bothered them during the previous two weeks, from 0 (*not at all*) to 3 (*nearly every day*). The measure has good validity and reliability [40]. In the present study, Cronbach’s alpha was .90.

### Procedure

The study protocol was approved by the Sponsorship and Registration Committee at the University of Liverpool (UoL000923). The study also had ethical approval from the National Health Service (NHS), Research Ethics Committee (NRES, 13/LO/0438) and the NHS Trust Research Governance Committee. The participation information sheets and consent form procedures used in the study were approved by these same sponsorship and ethics committees. Informed written consent was obtained from each participant who volunteered to participate in the study. Written consent was obtained prior to testing by the researcher (CO). Participation was entirely voluntary and participants were informed that they could freely withdraw from the study at any time. Consent also required that participants understood the study information and had the opportunity to address any questions they may have concerning the research. In accord with British Psychological Society (BPS) Research Ethics [41] all participants were of an age and competence to provide consent (16 years and older). The study was conducted in accordance with the British Psychological Society ethical guidelines and the principles expressed in the Declaration of Helsinki. Depressed patients were tested individually in the clinic, whereas control participants were tested individually at home (*n* = 48) or at a university location (*n* = 3) according to preference. Following consent, participants completed the written fluency task, the Goal Task and made ratings on goal importance and goal expectancy. Next, they completed the Goal Adjustment Scale and the PHQ-9. Participants completed further self-report and behavioural measures designed to address alternative research questions that are not discussed here.

### Power analysis

A post-hoc power (sensitivity) analysis revealed that the minimum between-groups effect size detectable with power of .80, a two-tailed alpha of .05, and the sample size recruited was *d* = .59. Using the same parameters, this study achieved power of .66 to detect a medium-sized difference (*d* = .50) between the depressed and control groups.

## Results

Data screening revealed that there were two extreme outliers (*z*s > 3.29) for mean approach goal expectancy in the depressed group and one extreme outlier in the control group. Following Tabachnick and Fidell [42], these univariate outliers were assigned a raw score on mean approach expectancy that was one unit more extreme than the lowest score. Henceforth, all study variables were normally distributed.

The depressed and control groups did not differ significantly on gender, *χ*^*2*^ < 1.1, age, *t* < 1, or the subjective importance of their goals *F*s < 1, all $\eta_{p}^{2}\text{s}<\;.01$. Clinical characteristics of the depressed group are reported in Table 1. Depressed participants showed variability in their previous experience of depression, therapy and whether they were currently taking medication. Most participants in the depressed group reported moderate to severe depressive symptoms.

### Idiographic approach and avoidance goals

Table 2 presents descriptive statistics for number of goals listed and mean goal ratings for each group (depressed *vs* control) with respect to each goal type (approach *vs* avoidance).

**Table 2:** Means and standard deviations (*SD*) for number, importance and expectancy of approach and avoidance goals, and goal disengagement and re-engagement after unattainable goals, in the depressed and control groups.

Group	Number		Importance		Expectancy		Diseng	Reeng
	App	Avd	App	Avd	App	Avd		
Depressed	5.21 (2.34)	4.93 (1.99)	7.68 (1.30)	7.70 (1.12)	6.24 (1.37)	5.49 (2.00)	11.07 (3.20)	17.40 (4.73)
Control	7.18 (2.72)	4.24 (2.12)	7.38 (0.89)	7.62 (1.62)	7.32 (1.08)	3.63 (1.93)	8.94 (2.57)	21.57 (3.93)

*Note*. App = approach goal, Avd = avoidance goal, Diseng = tendency to disengage from unattainable goals, Reeng = tendency to reengage with alternative goals when goals are thwarted.

A mixed ANOVA was conducted on number of goals with group (depressed vs. control) as a between-subjects factor and goal type (approach vs. avoidance) as a within-subjects factor. Results showed no significant main effect for group, *F*(1, 91) = 2.32, *p* = .13, $\eta_{p}^{2}\;=\;.02$. There was a significant main effect of goal type with participants listing more approach than avoidance goals, *F*(1, 91) = 42.51, *p* < .001, $\eta_{p}^{2}\;=\;.32$, qualified by the predicted significant interaction between goal type and group, *F*(1, 91) = 28.79, *p* < .001, $\eta_{p}^{2}\;=\;.24$.

As predicted, tests of simple effects showed that depressed participants generated significantly fewer approach goals than did control participants, *F*(1, 148) = 16.43, *p* < .001, *d* = .72 (see Table 2)**.** Counter to prediction, there was no significant difference between groups on number of avoidance goals, *F*(1, 148) = 2.05, *p* = .15, *d* = .33. Thus, compared to controls, depressed participants listed fewer approach goals but not more avoidance goals.

### Goal expectancy

The mixed ANOVA on mean goal expectancy with group as a between-subjects factor and goal type as a within-subjects factor showed no significant main effect for group, *F*(1, 91) = 2.34, *p* = .13, $\eta_{p}^{2}\;=\;.03$. However, there was a significant main effect of goal type, *F*(1, 91) = 94.61, *p* < .001, $\eta_{p}^{2}\;=\;.51$, qualified by the predicted interaction between group and goal type, *F*(1, 91) = 41.51, *p* < .001, $\eta_{p}^{2}\;=\;.31$.

As predicted, tests of simple effects revealed that, compared to controls, depressed participants judged their (desirable) approach goal outcomes as significantly less likely to happen, *F*(1, 180) = 10.09, *p* = .002, *d* = 1.00, and judged their (undesirable) to-be-avoided goal outcomes as significantly more likely to happen, *F*(1, 180) = 29.69, *p* < .001, *d* = .96. Thus, for both goal types, depressed participants were more pessimistic than controls.

### Goal disengagement and re-engagement

Counter to prediction, depressed participants reported significantly greater inclination to disengage from unattainable goals than did control participants, *t*(91) = 3.56, *p* = .001, *d* = .83. As predicted, however, depressed participants reported lower ability to re-engage with new goals after goal blockage than did controls, *t*(91) = 4.64, *p* < .001, *d* = 1.06.

## Discussion

This study examined approach and avoidance goal fluency and expectancy, and goal flexibility in depression. As hypothesized, compared to controls, depressed participants reported significantly fewer approach goals (but not more avoidance goals), and reported more pessimism around goal attainment. Unexpectedly, depressed people reported a greater tendency to disengage from unattainable goals than never depressed persons, and (as expected) a lower tendency to engage in new goals. These findings provide the first evidence that depressed people show a pattern of greater pessimism allied to a tendency to disengage from goal pursuit (including new goals) in the face of obstacles.

The findings offer partial support for the theoretical view that depression is characterized by low approach motivation [4] and are consistent with past research indicating impaired approach goal motivation in depression [20] and non-clinical dysphoria [38]. Counter to two studies of clinical depression [17–18], which found no significant group difference, depressed individuals in the present study showed significantly greater difficulty in generating personally meaningful approach goals than did controls. However, the present study was more adequately powered to detect a medium-sized group difference. Consistent with theory [4], our results support the view that depression is characterized by diminished approach goal motivation. Impaired approach goal pursuit may limit opportunities to experience rewarding goal outcomes, reducing positive affect. The lack of significant group differences on avoidance goal pursuit is consistent with past research using a clinical sample [17]. Larger group differences on approach goals compared to avoidance goals may reflect the salience of depressed mood versus anxious mood among our depressed participants, given that depressed mood is associated with the BAS.

Replicating Dickson et al.’s [17] study, there was evidence for more pessimistic goal expectancies in our depressed sample. Lower expectancies may diminish the effort and commitment an individual mobilizes towards a rewarding goal outcome thus risking a self-fulfilling prophecy and an exacerbation of symptoms of depression and hopelessness. Pessimistic expectations about to-be-avoided goal outcomes may strengthen a sense of goal-related threat, failure and hopelessness in the face of negative possibilities. A heightened focus on avoidance goal outcome expectancy may simultaneously weaken approach goal motivation [43], thus limiting capacity to switch one’s focus to potentially rewarding goal outcomes

The study’s most innovative contribution was its examination of goal flexibility in depressed persons. We found no evidence to support assumptions from self-regulatory perseveration theory [24] and related theoretical perspectives suggesting that an inability to disengage from unattainable goals characterizes depression. Unexpectedly, depressed participants reported significantly *greater* disengagement from unattainable goals than did controls. Although not predicted theoretically, our results tend to parallel those observed in a study by O’Connor and colleagues who found that goal disengagement in the face of unobtainable goals was positively associated with suicidal thinking and attempts [22]. Based on our findings, it may be that clinically depressed individuals are particularly sensitive to potential goal failure, increasing their tendency to disengage when difficulties are encountered. Moreover, the expectancy judgements observed in this study suggest that depressed people may disengage because they have more pessimistic appraisals of overcoming obstacles to goal attainment. Although it has been argued that disengagement from unattainable goals may be adaptive [28–29] [44], premature disengagement due to lack of persistence may be maladaptive. Although the questionnaire asked participants whether they disengage when goals are literally unattainable, the impossibility of a particular goal is often not easily discernible in daily life. Thus, the observed group differences may reflect a tendency for depressed persons to give up prematurely when goal attainment is perceived to be unlikely, if not impossible. Alternatively, depressed persons may disengage behaviourally from unachievable goals but remain cognitively engaged due to the perceived importance of a goal resulting in painful goal engagement [32]. However, inter-item correlations revealed that items in the goal disengagement scale addressing cognitive versus behavioural disengagement were not empirically distinguishable in this study. Future studies should attempt to validate these findings using behavioural measures.

We found that depressed individuals also reported struggling to engage in alternative goals when they have to stop pursuing unattainable goals, mirroring the apparent difficulty depressed persons had in generating idiographic approach goals relative to controls. The relative inability to engage with new goals among depressed persons may also reflect a generalized pessimism around goal attainment. Reduced goal re-engagement is consistent with the view that individuals who have difficulty developing new plans would be at a greater risk of depression [45]. The results also point to the combination of high goal disengagement and low goal re-engagement as characteristic of depression. Interestingly, a study by O’Connor and colleagues [23] found that for older adults who had attempted suicide, high goal disengagement was related to a greater probability of self-harm at follow-up when participants were also low in goal re-engagement. However, the reverse relationship was found for young adults, suggesting complexities in the relationship between goal flexibility and self-harm. Future studies could examine whether difficulty re-engaging in pursuit of new goals, and an increased tendency to disengage in unattainable goals, represents a self-regulatory vulnerability marker for clinical depression and whether this marker applies in different life stages.

In terms of clinical implications, our results suggest that depressed persons struggle to articulate positive goals to strive for and find it more difficult to engage in alternative goals when existing goals become unattainable. These findings also suggest that depressed people have reduced expectancies for attaining goal outcomes. Although these results are cross-sectional and do not in themselves identify treatment targets, they indicate that there may be value in helping depressed people to identify approach goals to strive for and challenge pessimistic reasons why participants think that goals cannot be achieved. In this respect, the results are consistent with behavioural activation, which encourages depressed persons to engage behaviourally with goals in the world, and cognitive-behaviour therapy, which may be useful in tackling hopelessness around goal attainment. Interventions that specifically address goal pursuit have shown promise in reducing depressive symptoms in clinical and non-clinical populations. Building confidence and self-efficacy around goal pursuit may also be a useful strategy in preventing depressive onset, but further research is needed.

Some methodological considerations deserve comment. Diagnosis was determined via self-report (PHQ-9), clinical assessment and a brief structured interview. In contrast to the brief interview schedule designed for this study, the use of a validated interview schedule such as the SCID would provide a more comprehensive assessment. There were two 16-year-old participants in the otherwise adult sample, but the pattern of significant results was identical when these two participants were excluded. Furthermore, the cross-sectional design means that it is unclear whether depression causes dysregulation of goal processes or whether dysregulated goal processes cause depression, or whether goal dysregulation is epiphenomenal to the depressive episode. Longitudinal studies could usefully clarify whether dysregulated goal pursuit precedes depressive episodes. Future research would benefit from supplementing self-report measures of goal flexibility with behavioural measures that capture goal-directed effort among alternative pursuits.

In conclusion, our findings illustrate altered goal processes in depression, characterized by blunted approach goal motivation, pessimistic goal expectancies, and increased goal disengagement and reduced goal re-engagement in the face of goal difficulties. Notably, effect sizes for group differences were large [46]. Although these findings cast doubt on the notion that depression is characterized by an inability to disengage from unattainable goals, future studies using triangulating measures should examine whether the self-reported tendency to disengage from unattainable goals in depression reflects genuine behavioural and/or cognitive disengagement.

## Supporting Information

S1 File SPSS datafile for study variables. (SAV)
